# Enhanced Microwave Deicing Capacity of Cement Pavement with Carbon Fiber Screens

**DOI:** 10.3390/ma17071488

**Published:** 2024-03-25

**Authors:** Jiangjiang Li, Peng Zhao, Minghai Jing, Xiao Luo, Jiaqi Guo, Fei Zhang

**Affiliations:** 1 School of Materials Science & Engineering, Chang’an University, Xi’an 710061, China; lijiang_s@yulinu.edu.cn (J.L.); jingmh@nxu.edu.cn (M.J.); 13981203573@163.com (X.L.); gjq18624951231@163.com (J.G.); 2 School of Energy Engineering, Yulin University, Yulin 719000, China; 3 Civil Engineering Department, School of Architecture and Engineering, Yulin University, Yulin 719000, China; zhangfei@yulinu.edu.cn; 4 Yulin HDPE Double-Wall Corrugated Pipe Engineering Technology Research Center, Yulin 719000, China

**Keywords:** carbon fiber screen, deicing, microwave deicing, structure absorbing, cement concrete pavement

## Abstract

The combination of an absorbing structure and a road is a promising strategy for road deicing using microwaves. In this study, cement mortar (CM) specimens containing a carbon fiber screen (CFS) were prepared to concentrate electromagnetic losses on a road surface. The effect of the size and depth of the CFS on the surface heating efficiency of the microwave was studied and optimized, and a microwave deicing experiment was conducted. The results indicated that the destructive interference produced by the CFS led to the effective surface heating of the CM/CFS specimens. The optimal surface heating rate was 0.83 °C/s when the spacing, depth, and width of the CFS were 5.22, 13.31, and 2.80 mm, respectively. The deicing time was shortened by 21.68% from 83 to 65 s, and the heating rate increased by 17.14% from 0.70 to 0.82 °C/s for the specimen with CFS-1, which was 15 mm depth. Our results demonstrate that CM/CFS composite structures can be effectively applied to increase the capacity and accelerate the development of the microwave deicing of roads.

## 1. Introduction

In recent years, materials that strongly absorb microwaves have been added to or have replaced the materials in roads to enhance the ability to deice these roads with microwaves [1,2,3,4,5,6]. Among these materials, layered pavement combining microwave-absorbent materials and structures has shown excellent deicing performance [7,8,9,10,11]. Therefore, combining microwave-induced deicing pavement with microwave-absorbing structures has broad application prospects.

Resonant-type absorption structures, in which a reflective layer is embedded, use the interference of electromagnetic waves to achieve microwave absorption [12,13,14]. Compared with metal layers, flexible frequency selective surfaces, such as carbon fiber screens (CFSs) and glass fiber screens (GFSs), have been widely used in the electromagnetic wave absorption and shielding of building materials [15,16].

In particular, considerable research and discussions have focused on absorption structures based on CFSs. Zhao et al. [17] buried inductively activated carbon fiber and two sub-carbon fibers arranged vertically in polyurethane resin, forming a composite. The reflected electromagnetic wave’s phase and amplitude were separately controlled by each subregion, where adjusting the materials could minimize the final resultant vector (final reflectivity). Zhang et al. [18] designed and fabricated carbon fiber/epoxy composites with grid structures considering gradient impedance (impedance gradually changes). The composites with three-layer grid structures showed broadband microwave absorbing properties with reflectivity values of less than −10 dB in the range of 10–18 GHz. Shah et al. [19] studied the effect of arrayed carbon fibers in epoxy resin on microwave absorption. They found that the strong conductivity of perpendicularly cross-linked carbon fibers resulted in poor microwave absorption performance. Li et al. [20] prepared interference microwave-absorbing cermet composites with steel wire mesh screens. The microwave interference owing to the screens substantially enhanced the microwave-absorbing performance of the composites. In addition, the CFSs embedded in a road increased the road performance and bearing capacity of concrete pavement under freeze–thaw cycles. Lai et al. [21] buried carbon fiber grille in airport cement concrete pavement to investigate conductive snow-melting, which indicated the CM/CFS composite could meet road performance. Haghnejad et al. [22] investigated the effect of freeze–thaw cycles on the behavior of roller-compacted concrete pavement (RCCP) reinforced with recycled polypropylene fibers. Their results indicated that specimens reinforced with fiber had better performance than the other RCCPs after freeze–thaw cycles. However, investigations into applying CFSs to improve the surface heating or deicing of roads are lacking.

Therefore, in this study, cement mortar (CM) specimens containing CFSs were prepared. First, the effect of the size and depth of the CFS on the surface heating efficiency was studied. Then, a microwave deicing experiment was conducted. Subsequently, the size and depth of the CFSs were optimized to achieve a maximum surface heating rate (SHR). Finally, the microwave losses were discussed from the perspective of the interaction between microwaves and CFSs and the effect of CFSs on microwave propagation. The results are valuable for improving the deicing of pavement containing CFSs.

## 2. Materials and Methods

### 2.1. Materials

Ordinary Portland cement produced in Baoji, Shaanxi, was used, and the properties are shown in Table 1.

Natural sand was produced in Xi’an, Shaanxi, and the properties are shown in Table 2. Tap water was used to mix with the cement. The base polyacrylonitrile (PAN) CFS was purchased from Shanghai Xiao Que Industrial Co., Ltd. (Shanghai, China).

**Figure 1:**
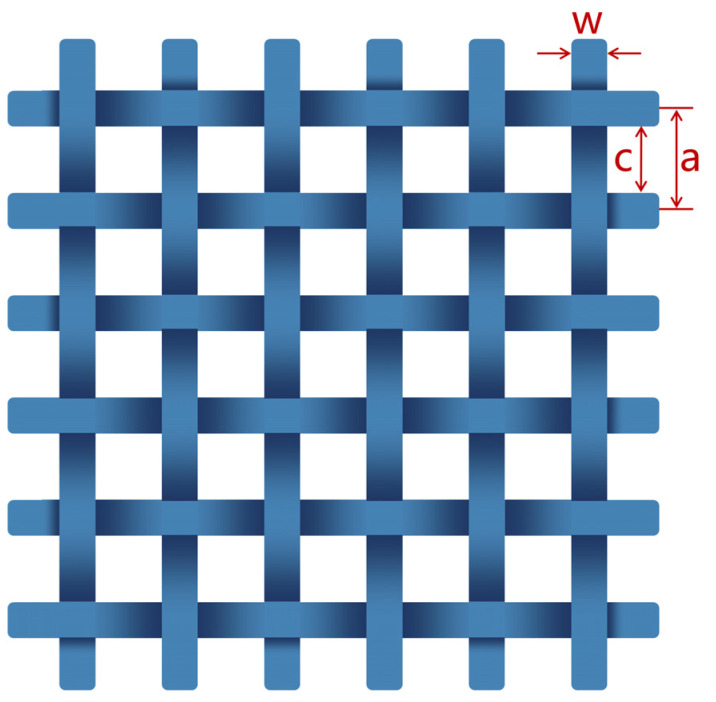
Schematic diagram of the CFS.

Figure 1 shows a schematic diagram of the CFS, where parameters a, c, and w represent the periodicity, spacing, and width of the CFS, respectively.

The parameters of different types of CFSs are shown in Table 3.

### 2.2. Methods

#### 2.2.1. Preparation of Composite Specimens

**Figure 2:**
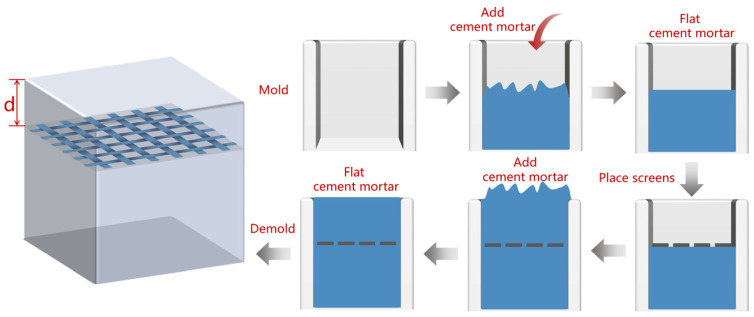
Schematic of the preparation of the CFS/CM composites.

The CFS/CM composite specimens were prepared by embedding a CFS in the CM (cement/water/dry sand = 1:0.4:2.5). The scheme and preparation of the CFS/CM composites specimens are shown in Figure 2, where d represents the depth of the CFS in the CM.

With the help of a shaking table, the cement mixture was poured into a steel mold with dimensions of 70.7 × 70.7 × (55.7~65.7) mm. Then, the CFSs with dimensions of 70 × 70 mm were embedded into the flat cement mixture. Finally, the CFS/CM specimens cured at 20 °C for 7 days were removed and wiped dry for testing. According to the type and depth of the CFS, 6 groups of specimens (labeled A to F) were prepared. The specific type and depth of the CFS/CM composite specimens are shown in Table 4.

#### 2.2.2. Temperature Test

**Figure 3:**
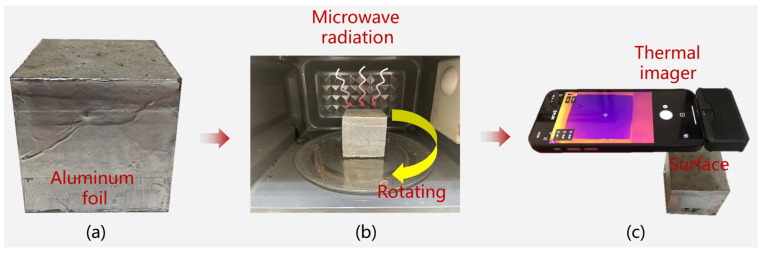
Acquisition of the temperature of the specimens: (**a**) package specimens, (**b**) microwave heating, and (**c**) temperature acquisition.

The radiation source was a microwave oven whose frequency and input power were 2.45 GHz and 700 W, respectively. The temperature acquisition process is shown in Figure 3.

Before heating, heat-insulating aluminum foil was pasted on the side and bottom of the specimens to prevent electromagnetic wave radiation, as shown in Figure 3a [23]. In addition, the specimens were kept in the furnace for 6 h to ensure a constant temperature of 25 °C. During heating, the rotating glass plate eliminated the error caused by uneven radiation, and the rotating speed was 4 r/min, as shown in Figure 3b. The temperature was recorded using a FLIR ONE PRO thermal imager (Teledyne FLIR, Portland, OR, USA) with a thermal resolution of 19,200 pixels (160 × 120), as shown in Figure 3c. Note that only one temperature recording could be obtained for each heating process, and the temperature needed to be remeasured for each process. The surface temperature was measured after 0, 30, 60, 90, and 120 s of radiation.

Due to the uniform heating and thermal insulation boundary of the side and bottom, the internal temperature was acquired by measuring the side temperature obtained by riving the aluminum foil, and the temperature was measured after 0 and 15 s of radiation.

#### 2.2.3. Microwave Deicing Test

**Figure 4:**
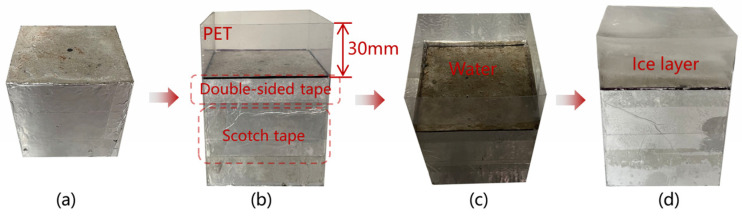
Process of icing the specimens: (**a**) specimen, (**b**) preparation fence, (**c**) adding water, and (**d**) ice on specimen.

**Figure 5:**
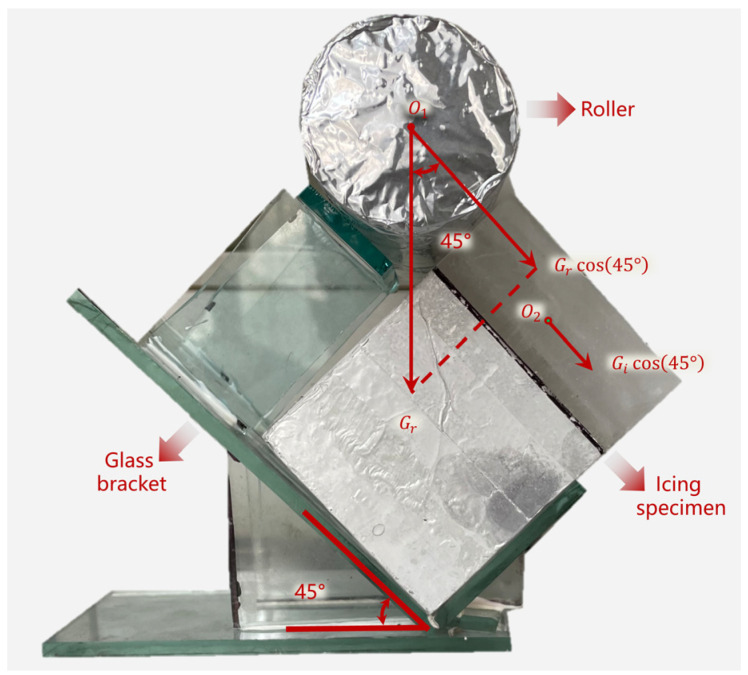
Microwave deicing device used in the laboratory.

Figure 4 and Figure 5 show the method used to freeze ice on the specimens and the deicing devices that were used in the laboratory to replicate the microwave procedure of deicing vehicles.

As shown in Figure 4, the method used to freeze ice on the specimen surface was as follows: First, the polyethylene terephthalate (PET) was fixed around the specimen with double-sided adhesive and scotch tape and kept 30 mm beyond the surface. Second, the specimens were placed in a −30 °C freezer. Third, tap water was added three times over 60 min to ensure a smooth ice surface. Finally, the icing specimen was complete, as shown in Figure 4d, when PET was removed after 24 h.

The icing specimens were immediately placed in the microwave oven with a deicing device after being removed from the chamber. The deicing device shown in Figure 5 is composed of a support part (glass support), an external force part (roller), and an iced specimen. The gravity of the roller (Gr), which was wrapped with aluminum foil, could be changed by adding or removing the steel ball inside. The tangential force of the ice layer was composed of the ice layer’s partial force and the roller’s gravity.

To simulate a real road deicing circumstance in the laboratory, the relevant parameters of deicing vehicles were applied. According to the ice-breaking roller structure in reference [24], the shear force on the single-chip milling cutter was

(1) $$F_{\tau }^{\prime }=\tau A=\tau \mathrm{hd}$$

where $F_{\tau }^{\prime }$ is the shear force N of the single-chip milling cutter; τ is the shear stress, which was 0.45 MPa in this study; A is the force area of the single milling cutter, m^2^; h is the width of the single milling cutter, 60 mm; and d is the thickness of the milling cutter, 0.8 mm.

The transverse shear force on the ice layer is

(2) $$F_{\tau }=N\times F_{\tau }^{\prime }$$

where N is the number of milling blades on the ice breaking roller, which was 10 in this study.

The roller mass is

(3) $$m=\frac{0.07\times F_{\tau }/L-\rho Vg\sin\varphi }{g\sin\varphi }$$

where L is the width of the ice-breaking roller, the size was 2000 mm; ρ is the density of the ice layer (800 kg/m^3^); V is the volume of the ice layer (0.147 × 10^−3^ m^3^ for a thickness of 30 mm); g is the acceleration due to gravity (9.8 N/kg); φ is the angle between the ice surface and the level in the deicing device, which was 45°; and m is the mass of the roller. The mass of the roller was 1.07 kg by calculation. When the ice fell off, the heating time was recorded as the microwave deicing time (MDT).

#### 2.2.4. Optimization of the Surface Heating Rate (SHR)

Microwave deicing is a complex process that depends on many factors, such as initial and ambient temperatures, ice thickness, electromagnetic properties of the materials, and microwave frequency, which directly or indirectly affect deicing efficiency [25]. In order to anticipate and optimize the SHR, which is an important index used to gauge microwave deicing efficiency, as the SHR depends on many factors, the right mathematical model must be chosen. Response surface methodology (RSM) is a mathematical technique used to combine experimental data with those from a mathematical model for application in optimization, forecasting, or interpretation [26]. RSM produces results that are more in line with the actual working conditions because it is established using experimental results. In addition, RSM is a multivariate statistical technique that permits both the description of the effects of independent components in the process and a large decrease in the number of experiments that must be conducted [27].

**Figure 6:**
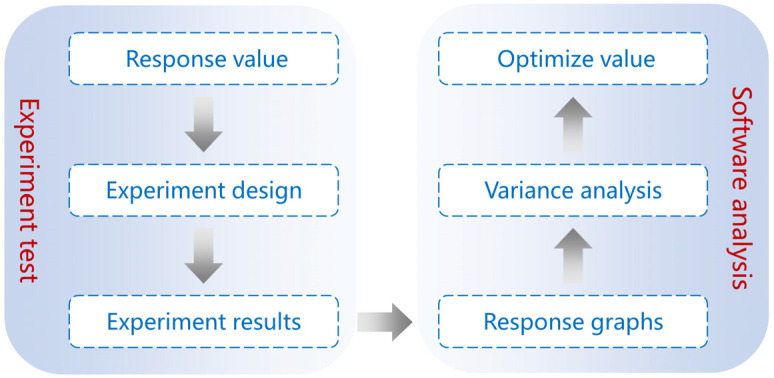
Process of optimizing parameters using RSM.

The process of optimizing the size and depth of the CFSs using RSM was divided into two parts, including experimental test and soft analysis, as shown in Figure 6.

In the experimental test part, the Box–Behnken (BB) design procedure, which is a three-level incomplete combination of many factors that can be used instead of a labor-intensive full-factorial design and is easily accessible through Design Expert 12.0 (Stat-Ease, Minneapolis, MN, USA, was used to design the experiment using the space, width, and depth of the CFS as variables [28]. The coded values of the variables and experiment results used in the BB design are shown in Table 5 and Table 6, respectively.

In the soft analysis part, the model equation and response graphs were obtained from experimental results. Then, an analysis of the variance model was used to validate the accuracy of the model. Finally, the optimal variables for the CFS were obtained using the model.

## 3. Results

### 3.1. The SHR of the CFS/CM Composite Specimen

The aim of microwave deicing is to transform electromagnetic energy into thermal energy via the microwave-absorbing materials in the pavement and then transmit that energy to the interface between the pavement and the ice layer, diminishing the ice layer’s stickiness. Therefore, the thermal performance of the interface between the specimen and the air is an important reference index for evaluating deicing performance. The influence of the size and depth of the CFSs on the thermal characteristics of a road surface was investigated.

#### 3.1.1. Effect of CFS Size on the SHR

**Figure 7:**
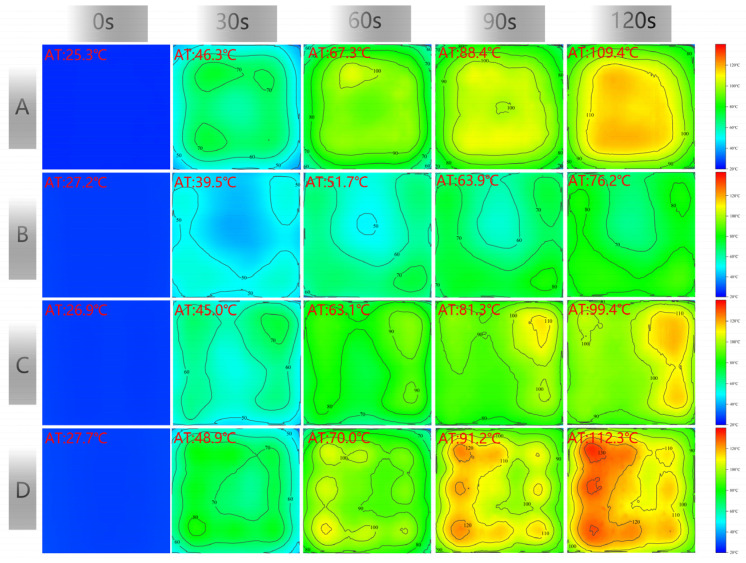
The surface temperature of the CFS/CM specimens, (**A**) without CFS, the types of (**B**–**D**) are CFS-1, CFS-2, and CFS-3, and depths are 5 mm. AT is the average temperature.

Figure 7 shows the surface temperature distribution of the CFS/CM specimens containing CFSs of different sizes after microwave heating. Sample A did not contain a CFS, whereas samples B, C, and D contained CFS-1, CFS-2, and CFS-3, respectively, at a depth of 5 mm.

We determined whether the CFSs enhanced or reduced the surface heating of the specimens by estimating the surface average heating rate, and the results are shown in Figure 7. In 0~120 s, the average heating rate of specimens A, B, C, and D was 0.70, 0.41, 0.60, and 0.71 °C/s, respectively. The heating rate of specimen D was higher by 1.34% and that of specimen B was lower by 41.43% than that of specimen A.

The surface temperature of specimen A without a CFS was uniformly distributed, as shown in Figure 7. As the radiation time increased, the surface temperature continued to rise, exhibiting a relatively high-temperature distribution feature in the central region, while the surrounding areas showed a lower-temperature distribution, with a larger proportion in the middle region.

The CFS in specimen B was 10 mm, 5 mm, and 5 mm in terms of period, space, and depth, respectively. Specimen B showed rapid heating at the four corners and slow heating in the center, whose proportion was relatively small. Although the temperature distribution of specimen C was similar to that of specimen B, the average temperature was much higher than that of specimen B. The width of the CFS was narrow, and its spacing was larger in specimen C.

As the width and space of the CFS increased, the temperature distribution of specimen D showed a nine-grid distribution, whose area of temperature increase corresponded with the CFS’s gap.

#### 3.1.2. Effect of CFS Depth on the SHR

**Figure 8:**
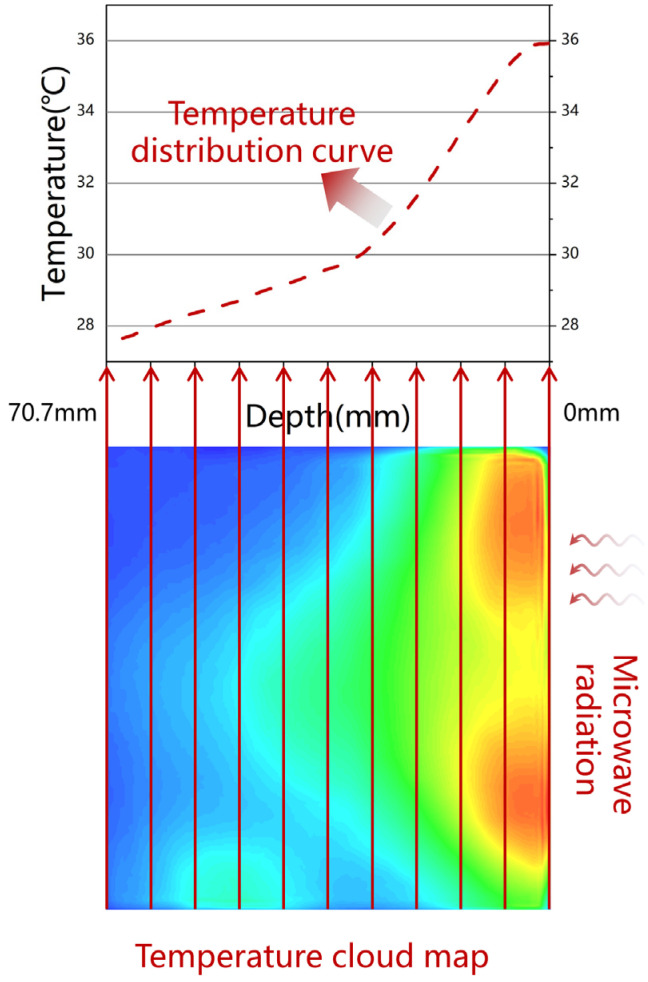
Acquisition of the temperature distribution in the radiation direction, temperature gradient from high temperature (red) to low temperature (blue).

Figure 8 shows the procedure for determining the temperature distribution of the specimens along the radiation path from the side temperature. The temperature cloud map, composed of thousands of temperature data points, was obtained by measuring the side temperatures of the specimens. Temperature data perpendicular to the radiation direction were accumulated and averaged, and the resulting value was used as the temperature data in the incident direction to obtain a continuous temperature distribution curve.

**Figure 9:**
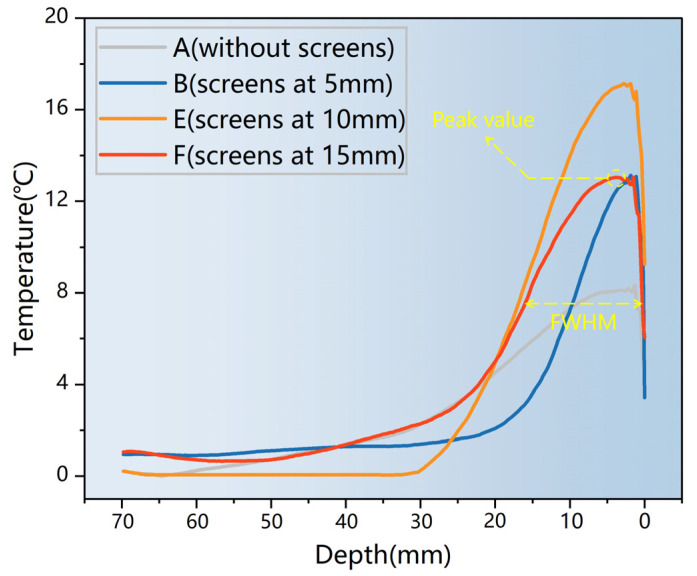
Temperature rise curves along the radiation direction.

The temperature rise curves derived from the method in Figure 8 after microwave heating for 15 s are presented in Figure 9. Specimen A had no screens; specimens B, E, and F contained screen CFS-1 at depths of 5, 10, and 15 mm, respectively.

As shown in Figure 9, all temperature rise curves exhibited a single peak, with a significant temperature drop near the surface, implying that thermal convection occurred between the surface and air. The peak value, position of the peak value, and full width at half-maximum (FWHM) of the longitudinal temperature distribution of the specimen are shown in Table 7.

The peak values of specimens A, B, E, and F were 8.19, 13.08, 17.13, and 13.05 °C, respectively, as shown in Table 7.

The peak value of E indicated its excellent heating ability: this specimen produced the best surface heating performance. Specimens B and E had similar peak values. The peak position of specimen B was 1.12 mm, which is closer to the surface, and its FWHM was narrowest at 10.63 mm. The narrow heating area prevented continuous heat transfer to the surface, resulting in specimen B having a lower surface heating capacity than specimen F, despite their similar peaks. The lowest peak was observed for specimen A [7].

### 3.2. Microwave Deicing Time

**Figure 10:**
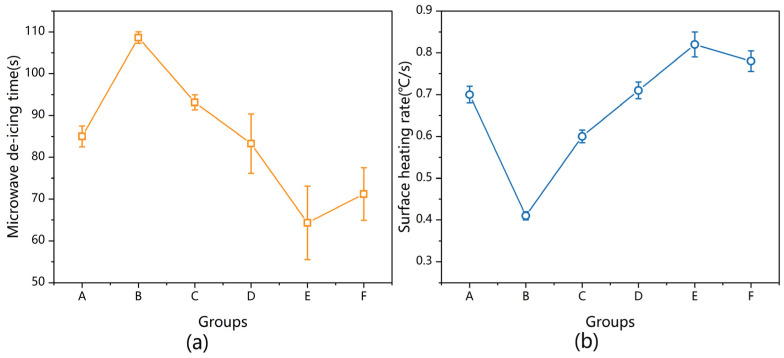
The relationship between (**a**) MDT and (**b**) the SHR.

Although the SHR can be used to characterize deicing performance, a deicing test was needed to verify the results, considering the differences in the thermal boundary conditions. Figure 10 shows the relationship between the MDT at −30 °C and the surface heating rate at room temperature. The deicing time was negatively correlated with the surface heating rate, as shown in Figure 10. The deicing time of specimen E was shortened by 21.68% from 83 s to 65 s, and the heating rate increased by 17.14% from 0.70 to 0.82 °C/s compared with the values of the control group.

The results of the reduction in the MDT in Figure 10 confirmed the improvement in deicing efficiency achieved by inserting a CFS into CM. Although the MDT increased in specimens B and C, this did not prevent the reasonable design to decrease the MDT. Therefore, these results suggested that deicing efficiency can be increased with the appropriate CFS/CM composite specimen.

The deicing efficiency ratio of specimen E, which was the deicing time relative to the control specimen, was 1.28. For magnetite cement concrete microwave de-icing pavement, in which magnetite is a widely studied road-absorbing material, the deicing efficiency ratio is 1.46~3.3 [29,30,31]. The improvement in the deicing efficiency of pavement with a CFS was lower than that of pavement with magnetite, while the CFS dissipated microwaves by the law of EM propagation, which is a new method that could be combined with absorbing materials to further improve deicing efficiency.

The strong agreement between the MDT at low temperatures and the surface heating rate at room temperature indicated that the complex deicing test could be replaced by a simple surface temperature test to characterize the deicing efficiency. Despite the differences in surrounding temperatures, heat conduction processes, reflectivity, and dielectric loss (the ice loss tangent was 0.0009 at −12 °C) between the MDT and the surface heating rate, these can be deemed insignificant in terms of relative comparison [32,33,34]. Only one initial temperature and one ice thickness were tested, which was found to be sufficient for demonstrating the deicing efficiency of pavement using the surface heating rate at room temperature to reduce the experimental cost.

### 3.3. Optimization of the SHR Based on RSM

As described in the previous section, the effects of the size and depth of the CFS on the SHR differed, and these factors interacted. Therefore, the best CFS size and depth were determined through RSM optimization.

#### 3.3.1. Variance Analyses

Equation (4) depicts the regression model of the surface heating rate response variable to factors A, B, and C, which were derived from the data in Table 6 using Design Expert 12.0 software.

P = 0.79 − 0.0025A + 0.0675B − 0.1AB − 0.015A^2^ − 0.13B^2^ − 0.01C^2^ (4)

where P is the surface heating rate and A, B, and C reflect the space, depth, and width of the CFS, respectively.

The equation’s correctness was statistically tested using analysis of variance (ANOVA), with the results shown in Table 8.

The polynomial parameter above was obtained by finding the minimum residual error. The primary goal of ANOVA is to identify relevant elements [35]. Because the F-value for independent variable B was the highest (10.41), this variable could be regarded as the main component influencing the surface heating rate. Notably, several of the factors had interaction effects (AB, AC, and BC).

**Figure 11:**
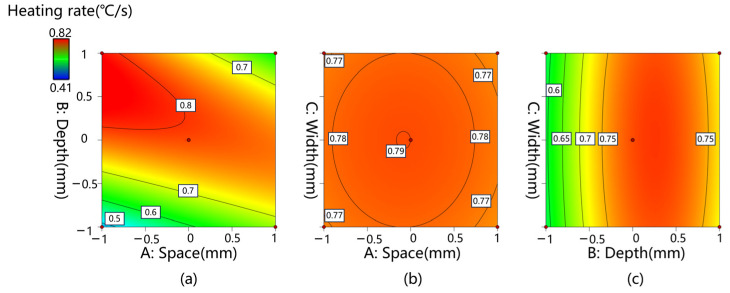
The SHR of the interactive effects of the variables, (**a**) Space and Depth, (**b**) Space and Width, (**c**) Depth and Width.

Figure 11 shows the 2D contour lines of the surface heating rate for different spaces, depths, and widths of the CFS.

Each contour curve indicates a combination of two measured variables, with the other remaining at zero. If a model includes variable interaction effects, the contour lines are curved rather than straight [36]. The plots in Figure 11a demonstrate that the surface heating rate was mostly determined by the interplay between space and depth, and the best combination was 5 mm and 15 mm, respectively. The ellipse graph in Figure 11b indicates that the best response value was located in the center band of the depth and width values. The approximate straight lines in Figure 11c imply that little interaction occurred between width and depth when the space was zero.

#### 3.3.2. Optimization of the SHR

The analysis of variance offers data on relevance as well as the fit correctness. In Table 8, the *p*-value of the model is 0.0254, which is less than 0.05, indicating the model is meaningful. The R-squared of Equation (4) is 0.8604, and the lack of fit (sum of squares = 0.183) indicates that the equation fitted the experiment values. Furthermore, the low pure error (0.000) indicates that the experimental data were reproducible. Therefore, the polynomial model above can be used, instead of the actual results, for optimization, predictions, or interpretation.

**Figure 12:**
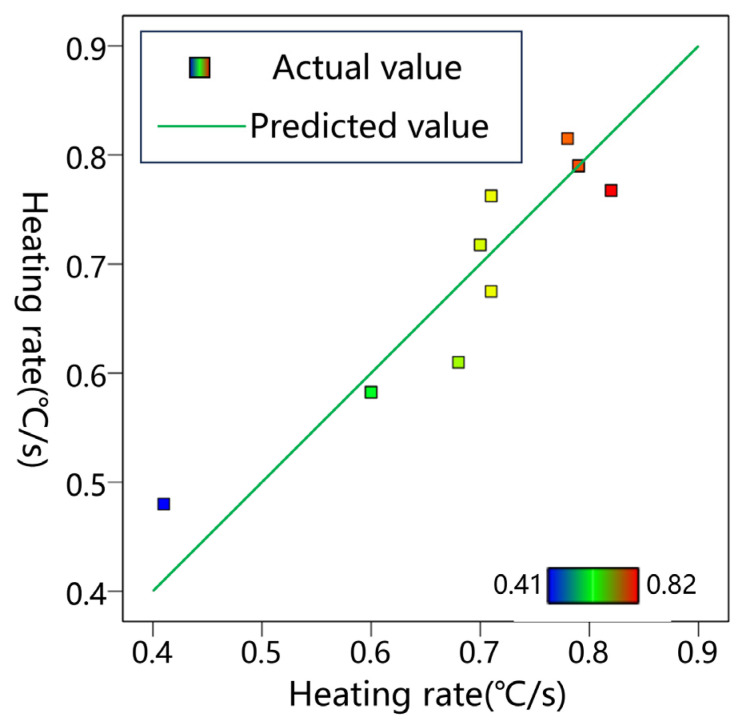
Correlation between the actual and predicted values.

Figure 12 shows the surface heating rate obtained from both experiments and models.

The predicted values strongly correlated with the experimental values, demonstrating that the model was capable of predicting the experimental results. Therefore, the values of each variable that maximize the surface heating rate were predicted by the model. The optimization of the CFS parameters were obtained directly through Design Expert 12.0 software, which were 5.223, 13.309, and 2.795 mm for space, depth, and width, respectively, and the optimal SHR was 0.829 °C/s.

## 4. Discussion

The reflection and absorption processes of CFSs were extensively studied and discussed. In this section, microwave losses are discussed from the perspective of the interaction between the microwaves and CFSs and regarding the effect of a CFS on microwave propagation.

### 4.1. Interaction between Microwaves and CFS

**Figure 13:**
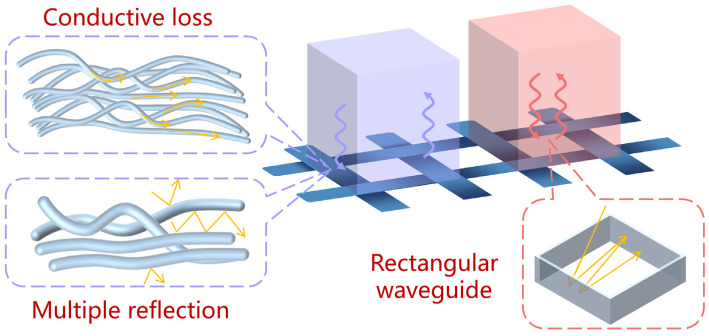
The interaction between electromagnetic waves and the CFS, the purple and pink arrows represent the interaction mechanisms between electromagnetic waves and CF bundles, as well as the space of CFSs, respectively.

The effects of the microwave loss and reflection mechanism on the CFS are shown in Figure 13.

The purple region in Figure 13 depicts the microwaves irradiated on the carbon fibers. Carbon fiber is a dielectric loss wave-absorbing material, which is lost from conductive loss and polarization relaxation [37]. Therefore, the induced current caused by the conductive network transforms the microwaves into Joule heat or other forms when the microwaves reach the carbon fiber. The microwaves are consumed in the CM because of the multiple reflections within the carbon fiber bundle and reflected into the CM because of the scattering outside the carbon fiber bundle. In other words, of the microwaves that radiated onto carbon fibers, part is absorbed by the carbon fibers and the CM in a gap, whereas the other part disperses in the CM, which produced a nine-grid surface temperature distribution on specimen D, as shown in Figure 7.

The pink region in Figure 13 is the microwaves irradiated on the gaps of CFS. In this case, one unit of the CFS was considered as a rectangular waveguide, in which the waveguide wall was a carbon fiber bundle and the medium within the waveguide was CM [11]. According to electromagnetic field theory, only when the operating frequency is higher than the cutoff frequency can electromagnetic waves propagate in a waveguide. The cutoff frequency is expressed as

(5) $$\omega_{C_{mp}}=\frac{1}{\sqrt{\mu \varepsilon }}{\left[{\left(\frac{m\pi }{a}\right)}^{2}+{\left(\frac{p\pi }{b}\right)}^{2}\right]}^{1/2}$$

where $\omega_{C}$ is the cutoff frequency, a and b are the dimensions of the waveguide, μ and ε are the material parameters of the medium inside the waveguide, and m and p are the number of modes. According to Equation (5), the modes and cutoff frequency can be determined using the dimensions of the waveguide when the medium is identified. Therefore, the size of a CFS completely determines whether electromagnetic waves may pass through it.

The reflectivity of a CFS can be determined using the equivalent impedance ($Z_{g}$), which is derived from Equations (6)–(9) [38]. The measured values of the relative dielectric constant ($\varepsilon_{r}$) of a CM of 6.623–0.674j at 2.45 GHz was adopted in the calculations.

(6) $$\varepsilon_{eff}=\left(\varepsilon_{r}+1\right)/2$$

(7) $$\alpha =\frac{ak_{eff}}{\pi }\ln\left(\frac{1}{\sin\frac{\pi w}{2a}}\right)$$

(8) $$\eta_{eff}=Z_{0}\sqrt{\frac{1}{\varepsilon_{eff}}}$$

(9) $$Z_{g}=j\frac{\eta_{eff}}{2}\alpha$$

where $\varepsilon_{eff}$ is the effective dielectric constant of a CFS in a CM; α is the screen parameters of the CFS; a and w are the screen sizes, as shown in Figure 1; $k_{eff}$ = $k_{0}\sqrt{\varepsilon_{eff}}$ and $k_{0}$ are the wave number in the CM and free space, respectively; $\eta_{eff}$ is the wave impedance of a CM with a CFS; $Z_{0}$ is the free space impedance of 377 Ω; and $Z_{g}$ is the equivalent impedance of a CM with a CFS. The equation shows that $Z_{g}$ is affected by $\varepsilon_{r}$, a, w, and f. If $\varepsilon_{r}$ and f of the specimen are known, the variance in the equivalent impedance of a CM with a CFS can be mostly ascribed to the mesh sizes of a and w, implying a different CFS would change the equivalent impedance of a CFS in a CM. The calculated values of the equivalent impedances were 10.68 Ω, 49.97 Ω, and 59.19 Ω for CFS-1, CFS-2, and CFS-3 in a CM at a 2.45 GHz frequency, respectively. The electromagnetic wave reflection loss at the interface of the CM and CFS was −1.27 dB, −6.16 dB, and −7.47 dB in specimens B, C, and D, respectively [39]. Consequently, the mismatch in the CM caused the CFS-3 reflection to be greatest.

### 4.2. Effect of CFS on Microwave Propagation

**Figure 14:**
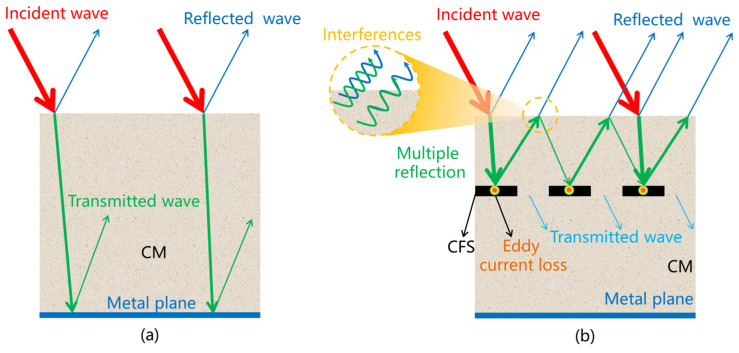
The interaction between electromagnetic waves and a CFS: (**a**) CM without CFS, (**b**) CFS/CM composite specimen.

Figure 14 shows the microwave propagation in the CFS/CM composite specimens. The incident microwaves penetrated the CFS/CM specimen with some reflection because of the surface impedance mismatch in the CM, as shown in Figure 14a.

Due to the lack of a CFS and the poor microwave absorption of CM, the majority of the microwaves entering the CFS/CM specimen without a screen propagated forward and backward after being reflected by the metal plate until being consumed by the CM. The microwaves were lost throughout the specimens because of the long loss route, which resulted in a wide heating area and low heating efficiency, as shown for the longitudinal temperature of specimen A in Figure 8.

The heating efficiency close to the surface can be increased by attenuating the microwave entering the CFS/CM specimen near the surface. The microwaves reached the surface of the CFS/CM specimens with screens reflected into the air because of impedance mismatch, as shown in Figure 14b. Multiple reflections between the CFS and the surface cause the incident microwaves to be lost, which increases the heating efficiency close to the surface. Multiple reflecting microwaves penetrating the surface return to the air (green wave arrow) and interfere with the reflected microwave (blue wave arrow) on the surface. Constructive interferences increase the microwave’s amplitude, increasing the surface loss and heating ability, whereas destructive interferences completely convert the microwave into heat or other forms, substantially increasing the surface heating capacity. The depth of the CFS determines whether and what kind of interference happens, whereas the size of the CFS determines the amount of interference.

According to interference theory, an electromagnetic wave is strongly absorbed when the distance d between two reflecting surfaces and the wavelength λ of the incoming wave fulfills Equation (10) [40]:

(10) $$d=\left(2n+1\right)\frac{v_{c}}{4f_{m}\sqrt{\varepsilon_{r}\mu_{r}}}n=0,1,2,\cdots$$

where $\varepsilon_{r}$ and $\mu_{r}$ are the medium’s relative permittivity and permeability, respectively; $f_{m}$ is the absorption peak’s frequency; n is an integer; and $v_{c}$ is the speed of light in a vacuum. The 2.45 GHz microwave was extensively absorbed at d = 12.2 mm and n = 0, which is comparable to the optimal depth of 13.31 mm in Section 3.3.2. In conclusion, the effective surface heating of the CFS/CM specimens was mainly caused by the destructive interference produced by the CFS.

## 5. Conclusions and Recommendations

### 5.1. Conclusions

The surface microwave heating efficiency and deicing time of CFS/CM composite specimens were investigated, and the size and depth of the CFS were optimized using RSM with the objective of increasing the surface heating rate in this study. The following conclusions were drawn:

1. A combination of heating efficiency, heating position, and heating range resulted in the effective surface heating of the CFS/CM composite specimens.
2. The surface heating rate of the CFS/CM composite specimen could be used to characterize the deicing efficiency because the surface heating rate had a consistent negative correlation with the MDT.
3. The carbon fiber bundle of the CFS consumed some microwaves, and the size of the CFS determined the reflection and transmission of microwaves.
4. The effective surface heating of the CFS/CM specimens was mainly caused by the destructive interference produced by the CFS.

### 5.2. Importance and Further Studies

The approach used in his study is significant because it improves the pavement’s microwave heating capacity without adding absorbing materials by changing the pavement’s structure, and it shows the way ahead for raising the heating efficiency of pavement. Combining absorbing materials and structures will further improve the heating capacity of pavement.

The method of inserting a CFS into pavement reduces the dependence on absorbing materials, but it also poses challenges for construction because aggregates may potentially damage the integrity of a CFS. Further research studies should include the following: More deicing experiments should be conducted to quantify the exact deicing efficiency and surface heating rate for any condition in a statistically significant way. Long-term durability tests should be used to assess the practical longevity of CM/CFS composites. Electromagnetic exposure and carbon footprints should be considered to assess environmental impact. A broader range of CFS configurations should be tested to explore the parameter space fully for optimal deicing efficiency. Mechanical properties and the microscopic mechanism should be conducted by compression and bending tests after freeze–thaw cycles and scanning electron microscopy experiments.

## Figures and Tables

**Table 1:** The properties of ordinary Portland cement.

Properties		Values
Density/(g/cm^3^)		3.20
Average particle size/µm		20.80
Setting time/min	Initial	141.00
	Final	233.00
Flexural strength/MPa	3 d	3.80
	28 d	6.20
Compressive strength/MPa	3 d	18.90
	28 d	35.80
Main chemical composition/%	CaO	60.10
	SiO_2_	20.80

**Table 2:** The properties of sand.

Aggregates	Bulk Density/(g/cm^3^)	Apparent Density/(g/cm^3^)	Water Absorption/%	Soil Content/%	Fineness Modulus
Sand	1.54	2.36	0.58	0.60	1.8

**Table 3:** The parameters of different types of CFSs.

Type	Period (a)/mm	Space (c)/mm	Width (w)/mm
CFS-1	10	5	5
CFS-2	12	10	2
CFS-3	20	15	5

**Table 4:** The types and depths of CFS/CM composite specimens.

Group	A	B	C	D	E	F
Depth of CFS(d)/mm	/	5	5	5	10	15
Type of CFS	/	CFS-1	CFS-2	CFS-3	CFS-1	CFS-1

**Table 5:** Coded values of the variables used in the BB design.

Variable	Code	Levels		
		−1.0	0	+1.0
Space/mm	A	5	10	15
Depth/mm	B	5	10	15
Width/mm	C	2	3.5	5

**Table 6:** RSM experimental design and results.

No.	Variable			Response
	A	B	C	SHR/(°C/s)
1	0	0	0	0.79
2	0	0	0	0.79
3	0	0	0	0.79
4	0	0	0	0.79
5	0	1.0	−1.0	0.7
6	1.0	1.0	0	0.68
7	0	0	0	0.79
8	0	−1.0	1.0	0.6
9	−1.0	0	−1.0	0.82
10	−1.0	1.0	0	0.78
11	0	−1.0	−1.0	0.6
12	1.0	0	−1.0	0.71
13	−1.0	−1.0	0	0.41
14	1.0	−1.0	0	0.71
15	0	1.0	1.0	0.7
16	1.0	0	1.0	0.71
17	−1.0	0	1.0	0.82

**Table 7:** Peak value, position of the peak value, and the FWHM of the temperature rise curves.

Specimen	Peak Value/°C	Position of Peak Value/mm	FWHM/mm
A	8.19	2.05	21.5
B	13.08	1.12	10.63
E	17.13	2.8	15.68
F	13.05	3.55	17.17

**Table 8:** ANOVA for the quadratic response surface model.

Source	Sum of Squares	Degree of Freedom	Mean Square	F-Value	*p*-Value	Remark
Model	0.1511	9	0.0168	4.80	0.0254	significant
A	0.0000	1	0.0000	0.0143	0.9082	
B	0.0364	1	0.0364	10.41	0.0145	
AB	0.0400	1	0.0400	11.43	0.0117	
AC	0.0000	1	0.0000	0.0000	1.0000	
BC	0.0000	1	0.0000	0.0000	1.0000	
A2	0.0009	1	0.0009	0.2707	0.6189	
B2	0.0712	1	0.0712	20.33	0.0028	
C2	0.0004	1	0.0004	0.1203	0.7389	
Residual	0.0245	7	0.0035			
Lack of fit	0.0245	3	0.0082			
Pure error	0.0000	4	0.0000			
Cor total	0.1756	16	0.1756			

## Data Availability

Data are contained within the article.
